# Personalized statin therapy: Targeting metabolic processes to modulate the therapeutic and adverse effects of statins

**DOI:** 10.1016/j.heliyon.2025.e41629

**Published:** 2025-01-02

**Authors:** Zhuangqi Shi, Shuxin Han

**Affiliations:** Xinjiang Key Laboratory of Biological Resources and Genetic Engineering, College of Life Science and Technology, Xinjiang University, Urumqi, Xinjiang, 830046, China

**Keywords:** Statin, Adverse effects, Drug metabolism, Hepatic uptake, Drug disposition, Gene regulation, Transcription factors

## Abstract

Statins are widely used for treating lipid disorders and cardiovascular diseases. However, the therapeutic efficiency and adverse effects of statins vary among different patients, which numerous clinical and epidemiological studies have attributed to genetic polymorphisms in statin-metabolizing enzymes and transport proteins. The metabolic processes of statins are relatively complex, involving spontaneous or enzyme-catalyzed interconversion between more toxic lactone metabolites and active acid forms in the liver and bloodstream, influenced by multiple factors, including the expression levels of many metabolic enzymes and transporters. Addressing the variable statin therapeutic outcomes is a pressing clinical challenge. Transcription factors and epigenetic modifications regulate the metabolic enzymes and transporters involved in statin metabolism and disposition and, therefore, hold promise as 'personalized' targets for achieving optimized statin therapy. In this review, we explore the potential for customizing therapy by targeting the metabolism of statin medications. The biochemical bases of adverse reactions to statin drugs and their correlation with polymorphisms in metabolic enzymes and transporters are summarized. Next, we mainly focus on the regulatory roles of transcription factors and epigenetic modifications in regulating the gene expression of statin biochemical machinery. The recommendations for future therapies are finally proposed by targeting the central regulatory factors of statin metabolism.

## 1 Introduction

**Fig. 1:**
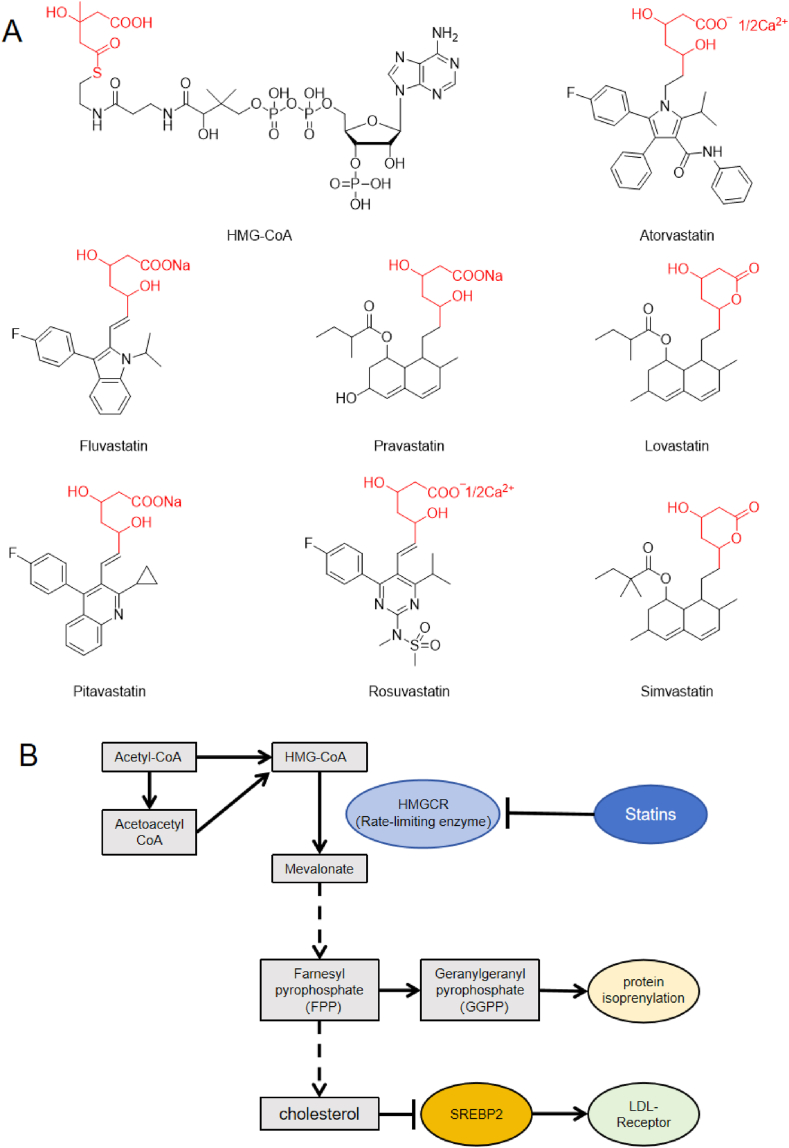
**Chemical structure and therapeutic mechanisms of statins.** A. A list of statin family members has a molecular structure similar to HMG-CoA. B. Statins inhibit the mevalonate pathway by competitively suppressing the rate-limiting enzyme HMGCR for cholesterol synthesis, thereby reducing cholesterol synthesis. In addition, reduced cholesterol synthesis upregulates the expression of LDL receptors, promoting the absorption of LDL in the liver. Inhibiting the mevalonate pathway can reduce protein isoprenylation, affecting various physiological activities. Unleashing the potential of statins: unraveling the intricacies of pleiotropy.

Cardiovascular diseases (CVD), including atherosclerosis, heart attacks, angina, and strokes, are the leading cause of death worldwide [1]. Elevated levels of low-density lipoprotein cholesterol (LDL-C) in the body represent a significant risk factor for the development and progression of cardiovascular diseases [2]. Hydroxy-3-methylglutaryl-coenzyme A reductase (HMGCR) is the first rate-limiting enzyme for the *de novo* synthesis of cholesterol, critically determining the cholesterol level in our body. HMGCR inhibitors, commonly known as statins, are currently the most effective and widely used oral medications for lowering cholesterol. As the cornerstone of lipid-lowering therapy, statins have been among the most extensively prescribed drugs globally for the past several decades [3]. The eight marketed statin types are lovastatin, simvastatin, pravastatin, fluvastatin, atorvastatin, pitavastatin, rosuvastatin, and cerivastatin which contain similar structures to hydroxy-3-methylglutaryl-coenzyme, A (HMG-CoA) (Fig. 1A). Lovastatin was initially isolated from cultures of *Aspergillus terreus* and became the first statin drug approved by the FDA in 1987 [4]. Simvastatin and pravastatin were developed through structural modifications of lovastatin, while fluvastatin was the first fully synthetic statin, structurally distinct from earlier semi-synthetic natural statins. Subsequent introductions included atorvastatin, cerivastatin, rosuvastatin, and pitavastatin, all fully synthetic statins. Cerivastatin was withdrawn in August 2001 due to a higher risk of rhabdomyolysis [5].

Despite the widespread use of statins, their adverse effects have increasingly become apparent. These adverse effects include liver toxicity manifested as elevated transaminase levels and cholestatic or mixed-type liver injury, as well as muscle toxicity presenting as myopathy, muscle pain, myositis, or rhabdomyolysis, and significantly impact the therapeutic efficacy and patient adherence to statin therapy [6]. Numerous studies highlight the crucial role of statin metabolism in determining their therapeutic effectiveness and adverse effects. This review summarizes statins' pharmaceutical mechanisms, adverse reactions, and metabolic processes, emphasizing new perspectives and unique significance in personalized statin therapy [[7], [8], [9]]. Our study aims to provide insights for optimizing future treatment strategies involving statins.

## 2 Therapeutic bases of statins

### 2.1 The lipid-lowering effect of statins

Cholesterol from dietary intake or endogenous synthesis participates in numerous physiological processes essential in forming cell barriers and signal transductions. Endogenous cholesterol is mainly synthesized in the liver and transported into the circulation via lipoproteins. Dysregulation of cholesterol metabolism can lead to hyperlipidemia resulting from an imbalance in cholesterol levels bound to lipoproteins with elevated LDL-C and decreased high-density lipoprotein cholesterol (HDL-C).

HMGCR is enriched in the liver and catalyzes HMG-CoA conversion to mevalonic acid, the rate-limiting enzyme in cholesterol synthesis. Due to their structural similarity to the HMG-CoA moiety, statins competitively inhibit HMGCR by binding to the enzyme and competitively preventing substrate binding (Fig. 1B). Inhibition of HMGCR activity disrupts HMG-CoA conversion to mevalonic acid, significantly reducing total cholesterol and LDL-C levels. The lowering of cholesterol synthesis levels by statins stimulates feedback mechanisms in the liver, leading to the upregulation of LDL receptors that enhances the uptake of LDL by the liver from the circulation, thereby reducing LDL-C levels in the bloodstream [6].

Moreover, the mevalonic acid pathway inhibition by statins decreases the production of protein isoprenylation, which are crucial for different cellular functions. That gives statins many effects beyond lipid-lowering, known as the pleiotropic effects of statins [10]. The effects include reducing inflammation [11], improving endothelial function [12], stabilizing atherosclerotic plaques [13], inhibiting thrombus formation [14], and anti-tumor effects [15].

### 2.2 The effect of statins on bone metabolism

Statins exert bone metabolic effects; many studies have reported that statins such as simvastatin, rosuvastatin, and atorvastatin can significantly reduce bone resorption and stimulate bone formation [[16], [17], [18]]. Current research has identified three main mechanisms by which statins affect bone metabolism: promoting osteoblast differentiation, inhibiting osteoblast apoptosis, and inhibiting osteoclast formation.

#### 2.2.1 Statins promote osteoblast differentiation

Statins cause a reduction in products of the mevalonate pathway, specifically farnesyl pyrophosphate (FPP) and geranylgeranyl pyrophosphate (GGPP), which are known to inhibit osteoblast differentiation [19]. The depletion of FPP or GGPP induced by statins can interfere with the isoprenoidation of GTP-binding proteins like Rho and Ras, thereby disrupting related signaling pathways. For instance, pirvastatin enhances the expression of bone morphogenetic protein-2 (BMP-2) and osteocalcin in human osteoblasts by inhibiting the Rho and Rho kinase pathways [20]. Simvastatin promotes osteoblast viability and differentiation through the Ras/Smad/Erk/BMP-2 signaling pathway [21,22]. Additionally, inhibiting protein isoprenylation leads to an upregulation of vascular endothelial growth factor (VEGF), which acts as a metabolic factor in osteoblasts by stimulating the production of phosphatidylinositol 3-kinase (PI3-K) [23,24]. Furthermore, lovastatin and simvastatin can induce osteogenic differentiation in periodontal ligament cells through the ERK1/2 pathway [25].

#### 2.2.2 Statins inhibit osteoblast apoptosis

Smad3, a key molecule in TGF-β signaling, plays a role in promoting bone formation. Recent research has shown that statins enhance the expression of Smad3 in MC3T3-E1 and UM-106 cells, thereby counteracting dexamethasone-induced osteoblast apoptosis in a dose-dependent fashion [26].

#### 2.2.3 Statins inhibit osteoclast formation

RANKL (receptor activator of NF-κB ligand), RANK (receptor activator of NF-κB), and osteoprotegerin (OPG) are crucial regulators of osteoclastogenesis, and statins can inhibit this process via the OPG/RANKL/RANK signaling pathway [27]. Specifically, statins inhibit osteoclastogenesis by reducing the expression of RANK and RANKL and increasing the expression of OPG [[28], [29], [30]]. In addition, atorvastatin inhibits osteoclast differentiation during IL-1β-induced osteoclastogenesis by downregulating NF-κB and MAPK signaling pathways [31].

On the other hand, Estrogen and estrogen receptor (ER) also play a significant role in inhibiting osteoclastogenesis. Estrogen reduces RANKL levels, thereby further suppressing osteoclast formation [32]. Studies have shown that simvastatin supports osteoblast survival and promotes differentiation by partially modulating the expression of estrogen receptor alpha (ERα), which helps treat osteoporosis associated with estrogen deficiency [33,34]. Simvastatin functions as an ERα ligand and co-activator, enhancing ERα-dependent transcriptional activity and promoting osteogenesis [35].

## 3 Pharmacokinetics of statins

The pharmacokinetic properties of statins are influenced by several factors, including their absorption, metabolism, lactone form or acid form, and hydrophilicity or lipophilicity [36]. Almost all statins are highly bound to serum proteins (about 90 %), mainly albumin [153], except for pravastatin, which has a binding rate of only about 50 %. Lovastatin and Simvastatin are first given as inactive lactone forms, which become active acid forms in the liver [5]. Statins like fluvastatin, pravastatin, atorvastatin, rosuvastatin, and pitavastatin are administered as active acid forms. Atorvastatin, simvastatin, lovastatin, and fluvastatin are classified as lipophilic statins, whereas pravastatin and rosuvastatin are more hydrophilic [6].

The metabolism of most statins primarily occurs in the liver, mediated by the three-phase endobiotic and xenobiotic metabolism (EXM) biochemical system [37]. Phase I reactions predominantly involve oxidation processes catalyzed by the cytochrome P450 (CYP450) enzymes [38]. Phase II reactions include conjugation processes such as glucuronidation, sulfation, and glutathionylation, respectively catalyzed by sulfotransferases (SULT), UDP-glucuronosyl transferases (UGT), and glutathione S-transferases (GST) [[39], [40], [41]]. Transport proteins mediate phase Ⅲ for absorption and excretion. The statin membrane transporters are categorized into two main superfamilies: solute carrier (SLC) transporters and ATP-binding cassette (ABC). The transporters responsible for statin uptake into cells belong to the SLC superfamily and include organic cation transporters (OCT), organic anion transporting polypeptides (OATP), and organic anion transporters (OAT). These transporters use pre-existing ion gradients as an energy source to move substrates and solutes in the same or opposite direction, thereby facilitating the membrane transport of various solutes (such as glucose, amino acids, and lipids) and contributing to maintaining cellular homeostasis [42]. Efflux transporter proteins belonging to the ABC superfamily utilize ATP hydrolysis energy to transport statins from the cytoplasm to the extracellular space, including P-glycoprotein (P-gp), multidrug resistance-associated protein (MRP), bile salt export pump (BSEP) and breast cancer resistance protein (BCRP) [43]. The biochemical characteristics, pharmacokinetics, drug interactions, adverse effects on tissues, metabolism, and transportation of statins are summarized in Table 1.

**Table 1:** Pharmacokinetics of statins.

Kind of statin	Atorvastatin	Fluvastatin	Pravastatin	Lovastatin	Pitavastatin	Rosuvastatin	Simvastatin
Clinical dosage/day	10–80 mg	20–80 mg	40–80 mg	20–80 mg	2–4 mg	5–40 mg	20–40 mg
Solubility	Lipophilicity	Lipophilicity	Hydrophilicity	Lipophilicity	Lipophilicity	Hydrophilicity	Lipophilicity
Form	Hydroxy acid	Hydroxy acid	Hydroxy acid	Lactone	Hydroxy acid	Hydroxy acid	Lactone
Metabolism places (from The highest to lowest)	Liver -intestine	Liver	Stomach-Kidney-Liver	Liver	Liver	Kidney - liver	Liver - intestine
Plasma half life (Hours)	14	1.2	1.8	3	11	19	2
Clearance	37.5 L/h	0.8 L/h/kg	0.810 L/h/kg	Not Available	43.4 L/h	28.3 L/h	31.8 L/h
absolute bioavailability	14 %	19–29 %	18 %	less than 5 %	51 %	20 %	less than 5 %
Adverse effects on tissues (from The highest to lowest)	gastro-intestinal symptom -liver disease -myopathy	gastro-intestinal symptom -Myopathy -liver disease	gastro-intestinal symptom -liver disease - myopathy	gastro-intestinal symptom - liver disease -myopathy	\	Gastro-intestinal symptom - liver disease - myopathy	Gastro-intestinal symptom - liver disease -myopathy
Drug Interactions (increase the risk)	Cyclosporine, anti-viral medications, azole antifungals, macrolide antibiotics, Grapefruit juice, Fibrates, Gemfibrozil, Niacin, colchicine	cyclosporine, fluconazole, Fibrates, Gemfibrozil, Niacin, colchicine	colchicine, fibrates, gemfibrozil, niacin, cyclosporine, Clarithro-Mycin, erythromycinmacrolide drugs (azithro-mycin)	itraconazole, ketoconazoleposaconazole, voriconazole, erythro-Mycin, clarithro-Mycin, telithromycin, boceprevir, telaprevir, nefazodone, cobicistat-containing products. CyclosporineGemfibrozil, fibrates, Niacin, Ranolazine, Colchicine, Amiodarone	Cyclo-sporine, Erythro-mycin, Rifampin, Gemfibrozil, Ffibrates, Niacin, colchicine	Cyclosporine, Teriflunomide, Enasidenib, Kamatinib, Fostamatinib, Febuxostat, Gemfibrozil, Ffibrates, Niacin, Simeprevir, Atazanavir, Ritonavir, lopinavir, ritonavir,	itraconazole, ketoconazole, posaconazole, voriconazole, erythromycin, clarithromycin nelfinavir, ritonavir, darunavir, ritonavir, boceprevir, telaprevir, cobicistat-containing products, nefazodone. cyclosporine, danazol, gemfibrozil, Fibrates, Daptomycin, Colchicine, Niacin, amiodarone, dronedarone, ranolazine, calcium channel blockers, Lomitapide, Grapefruit juice
Drug Interactions (stimulatory)	rifampin	\	bile acid chelators (cholestyramine, colestipol)	Take with food	\	aluminum hydroxide, magnesium hydroxide, antacids	\
Phase I Metabolic Enzyme	CYP3A4, CYP3A5	CYP2C9, CYP3A4, CYP2C8, CYP2D6	\	CYP3A4	CYP2C9	CYP2C9	CYP3A4, CYP3A5, CYP2C8, CYP2C9, CYP2D6
Phase Ⅱ Metabolic Enzyme	UGT1A1, UGT1A3	Not Available	\	UGT1A1, UGT1A3	UGT1A3, UGT2B7	UGT1A1, UGT1A3	UGT1A1, UGT1A3
Uptake Protein	OATP1B1, OATP1B3, OATP2B1	OATP1B1, NTCP	OATP2B1, OATP1B1	OATP1B1	OATP1B1, OATP1B3, NTCP	OATP1B1, OATP1B3, OATP2B1	OATP1B1
Efflux Protein	P-gp, BCRP	MRP2	MRP2, BSEP, BCRP	P-gp	MRP2, BCRP, P-gp	BCRP, P-gp	P-gp

**Fig. 2:**
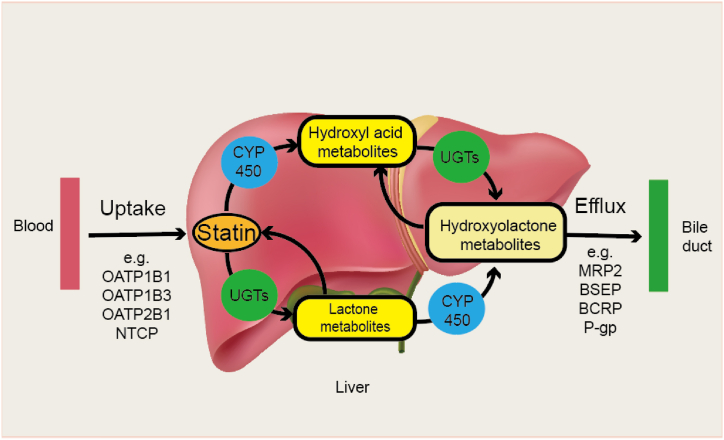
**Metabolism and transportation of statins.** Liver uptake transporters take statins from the blood into hepatocytes. In addition, lipophilic statins can be passively transported into hepatocytes. OATPs play an essential role in the uptake of statins to the liver. The metabolic processes of statins include phase I oxidation of CYP450 isoenzymes and phase II glucuronidation mediated by UGT isoenzymes. Glucuronide intermediates are unstable and rapidly decay into lactone forms. Lactone and acid forms can be converted to each other. Statins and their metabolites are excreted to the bile by efflux transporters, including MRP 2, BSEP, BCRP, and P-gp, expelling into the bile duct.

Lipophilic statins can quickly enter cells through passive diffusion and OATP, leading to higher exposure levels in non-hepatic tissues (Fig. 2). In contrast, hydrophilic statins are predominantly taken up via OATP, demonstrating higher liver selectivity [44]. Except for pravastatin and pitavastatin, most statins are metabolized in the liver by cytochrome P450 enzyme of gluconic aldehyde reaction mainly determines the metabolism of pravastatin, and the intervention of CYP3A enzyme is very little. Lactonization is an intermediate step in statin metabolism, including non-enzyme-catalyzed lactonization at low pH and UGTs-catalyzed formation of glucuronide intermediates, which are unstable and rapidly decay into lactone forms [45]. Lactone and acid forms can be converted to each other, and after hepatic metabolism, most are eliminated via bile, and a small portion is excreted via renal pathways ending in urine [46].

### 3.1 Atorvastatin

The clinically recommended starting dose for atorvastatin in clinical practice is 10 mg–20 mg once a day, with a dosage range of 10–80 mg and no time requirement for taking the medication. Atorvastatin undergoes extensive first pass metabolism in both the intestinal wall and the liver, leading to an absolute oral bioavailability of 14 % [47]. Elimination half-life of atorvastatin is 14 h. The distribution volume for atorvastatin acid was 381 L. The registered total plasma clearance of atorvastatin is 37.5 L/h [48]. Atorvastatin is mainly metabolized in the liver. Atorvastatin is primarily taken up into hepatocytes via OATP1B1, with studies indicating the involvement of OATP1B3 and OATP2B1. Inside hepatocytes, atorvastatin can be metabolized by CYP3A4 and CYP3A5, forming 2-hydroxy atorvastatin and 4-hydroxy atorvastatin [49]. These metabolites retain activity, exhibiting *in vitro* inhibition of HMGCR similar to atorvastatin. Approximately 70 % of the circulatory inhibitory activity against HMGCR is attributed to active metabolites [48]. On the other hand, UGT1A1/UGT1A3 conjugates atorvastatin or hydroxylated atorvastatin carboxylic acid via glucuronidation to form acyl-glucuronide intermediates, which cyclize to form inactive lactone forms. These lactones can hydrolyze to their corresponding acids, maintaining a balanced presence [45]. In blood circulation, 2-hydroxy atorvastatin acid and 2-hydroxy atorvastatin lactone are the primary metabolites [48]. Atorvastatin lactone exhibits a significantly higher affinity for CYP3A4 in human liver microsomes compared to atorvastatin acid, indicating that The majority of acid-form metabolites found in human plasma are produced through the mutual conversion of lactone metabolites, with lactonization being a critical first step in atorvastatin metabolism [50]. Besides liver metabolism, atorvastatin is also metabolized in the intestine. Atorvastatin lactone shows significant metabolism in human intestine microsomes, with clearance rates at 20 % of those in human liver microsomes [51]. Atorvastatin and its metabolites are eliminated via biliary excretion into feces, primarily mediated by P-gp and BCRP [52].

### 3.2 Fluvastatin

The clinical dosage of fluvastatin ranges from 20 mg to 80 mg, taken once daily at bedtime. The elimination half-life of fluvastatin is 1.2 h. Although fluvastatin can be rapidly and almost completely absorbed (>90 %). Due to extensive first-pass metabolism, the 2–10 mg bioavailability of doses is only 19 %–29 % [53]. According to reports, the steady-state distribution volume of fluatorvastatin in the human body is 0.42 ± 0.06 L/kg [54]. The registered total plasma clearance of fluvastatin is 0.8 L/h/kg. Fluvastatin is mainly metabolized in the liver. Fluvastatin is a substrate for OATP1B1/SLCO1B1, although the liver uptake of fluvastatin remains constant among SLCO1B1 polymorphisms, suggesting that alternative pathways may exist [55]. The study by Greupink et al. found that fluvastatin is a substrate for Na^+^/Taurocholate cotransporting polypeptide (NTCP) [56]. However, NTCP-mediated drug uptake into hepatocytes is a minor pathway under normal conditions. Fluvastatin undergoes primary metabolism by CYP2C9 in the liver, yielding two hydroxylated metabolites and one N-deisopropylated metabolite, none contributing to its lipid-lowering effects. 6-hydroxyfluvastatin is the major metabolite, constituting 80 % of the metabolites generated by CYP2C9 [57]. CYP2C9 also produces N-deisopropylfluvastatin, while CYP2C9, CYP3A4, CYP2C8, and CYP2D6 form 5-hydroxyfluvastatin [54]. Less than 5 % of fluvastatin is present in their lactone forms [58]. When ingested orally, mediated by MRP2, fluvastatin is mainly excreted in feces as its metabolites [53,59].

### 3.3 Pravastatin

The clinical dosage of pravastatin ranges from 40 to 80 mg, taken once daily at bedtime. After oral administration of pravastatin, absorption occurs rapidly, with the time to reach the peak drug concentration (Tmax) being approximately 1 h.The reported mean absorption time is 2.4 h, the oral bioavailability is approximately 18 %, respectively, and the liver extraction rate calculated based on absorptivity and bioavailability was 47 % [60]. Elimination half-life of lovastatin is 1.8 h. The distribution volume of pravastatin in the human body is 0.44 L/kg [61]. The clearance rate of pravastatin typically falls within the range of 0.810 L/h/kg [62]. pravastatin is efficiently absorbed from the upper small intestine through proton-coupled carrier-mediated mechanisms. It is transported into the intestines and liver by the OATP2B1 and OATP1B1 transporters [[63], [64], [65]]. While other statins are mainly excreted as metabolites, pravastatin undergoes minimal hepatic metabolism, with most excreted unchanged in bile and urine. The significant metabolites arise from chemical degradation in the stomach instead of cytochrome P450 metabolism in the liver, and the pravastatin and its metabolites are primarily cleared by the liver and kidneys, with tubular secretion playing a central role in renal elimination [61]. Approximately 64 % of intravenous doses are excreted as intact drugs in urine and feces. As a metabolite, the isomer 3′-α-isopravastatin is present in large quantities in the plasma, urine, and feces [60]. The main excretion route of pravastatin is through the bile, facilitated by the efflux of MRP2, BSEP, and BCRP [[66], [67], [68]].

### 3.4 Lovastatin

The starting dose of Lovastatin is 20 mg per day, taken during dinner time. The recommended dosage range for lovastatin is 10–80 mg per day. Elimination half-life of lovastatin is 3 h. Lovastatin is administered as a prodrug lactone and hydrolyzes *in vivo* to its active form, lovastatin acid. Lovastatin shows low bioavailability (less than 5 %) after oral administration. Once absorbed, lovastatin targets the liver, and the hydroxy acid form of lovastatin is less efficient than lactone by liver extraction [69]. Lovastatin undergoes extensive first-pass metabolism and is taken up in the liver via OATP1B1 [70], and less than 5 % of oral doses of lovastatin reach systemic circulation as an active inhibitor. Only 25 % of the active metabolites of lovastatin consist of β-hydroxy acid, with the remainder comprising other metabolites mediated by CYP3A4, such as 6′-β-hydroxy, 6′-exomethylene, and 3′-hydroxy lovastatin [71,72]. Lovastatin lactonization is mediated by UGT1A1 and UGT1A3, although at levels one order of magnitude lower than other statins [45]. Lovastatin is primarily disposed of via feces after its hepatic excretion by P-gp [73].

### 3.5 Pitavastatin

The recommended dosage range for pitavastatin is 2 mg–4 mg per day. The maximum recommended dose is 4 mg once daily, and there is no time requirement for taking the medication. Elimination half-life of pitavastatin is 11 h. The oral absolute bioavailability of the pitavastatin is 51 %. After a single dose, pitavastatin's apparent mean oral clearance is reportedly 43.4 L/h [74]. Pitavastatin has a relatively high bioavailability compared to other statins; the observed effect could result from enterohepatic reabsorption in the intestines after the drug's initial absorption. The mean volume of distribution is approximately 148 L/kg. Pitavastatin is primarily taken up in the liver via OATP1B1, with involvement also from OATP1B3 and NTCP [[75], [76], [77]]. The CYP450 system has minimal involvement in the metabolism of the compound, with a slight contribution from CYP2C9 [78], and the drug is primarily excreted unchanged [79]. The major pathway of pitavastatin metabolism counts on glucuronidation by UGTs to form pitavastatin lactone in the liver. The primary metabolite of pitavastatin in human plasma is lactone, formed by UGT1A3 and UGT2B7 [80], although UGT2B7-mediated conjugation is rarely observed in other statins [45]. Pitavastatin is excreted by MRP2, BCRP, and P-gp [52,81], with BCRP polymorphisms not affecting the pharmacokinetics of the statin [75].

### 3.6 Rosuvastatin

Adults are advised to take rosuvastatin calcium tablets in a daily dose ranging from 5 mg to 40 mg, And there is no time requirement for taking the medication. The recommended initial dose is 10–20 mg, administered orally once a day. The maximum dose of rosuvastatin calcium tablets is 40 mg and should only be used in patients who have not reached the LDL-C target with a dose of 20 mg. The elimination half-life of rosuvastatin is 19 h. The absolute bioavailability of rosuvastatin is about 20 % [46]. Rosuvastatin has a total clearance of 28.3 L/h [82]. As the most hydrophilic among commercially available statins, about 90 % of rosuvastatin has been reported to be excreted in feces and 10 % in urine following oral administration. The mean volume of distribution is approximately 134 L/kg. The drug undergoes minimal metabolism *in vivo*, as indicated by a^14^C radiolabeling study showing 76.8 % of the dose excreted unchanged in feces, 4.9 % as N-desmethyl rosuvastatin, and 1.8 % as rosuvastatin-5s-lactone [83]. In the liver, rosuvastatin is taken up to hepatocytes via OATP1B1, OATP1B3, and OATP2B1 [84,85]. CYP2C9 then converts the compound to the metabolite N-desmethyl rosuvastatin. However, rosuvastatin pharmacokinetics remain unaffected when administrated with fluconazole, a potent CYP2C9 inhibitor [86]. Rosuvastatin undergoes lactonization via UGT1A1 and UGT1A3 [87], and BCRP and P-gp are primarily responsible for the biliary excretion of the drug [52,84,88].

### 3.7 Simvastatin

The recommended dosage range for oral simvastatin tablets is 20 mg–40 mg. The maximum recommended dose is 40 mg of simvastatin tablets once a day, taken during dinner time. Elimination half-life of simvastatin is 2 h. Simvastatin is administered as an inactive lactone and undergoes reversible hydrolysis to its active metabolite, simvastatin acid [89]. Simvastatin undergoes extensive first-pass metabolism in the gut and liver, with absolute bioavailability below 5 %. The total body clearance of simvastatin is 31.8 L/h [89]. The uptake of simvastatin acid is predominantly mediated by OATP1B1, with genetic variations in OATP1B1 shown to affect simvastatin pharmacokinetics [90]. Simvastatin is mainly metabolized in the liver. Oxidative metabolism in the liver is primarily mediated by CYP3A4 and CYP3A5, with minor contributions from CYP2C8 and CYP2C9, producing metabolites such as 6′-hydroxy, 6′-hydroxymethyl, and 6′-exomethylene metabolites [91]. Clinical studies suggest that polymorphisms in CYP2D6 play a significant role in the efficacy and tolerance of simvastatin [92]. Although UGT1A1 and UGT1A3 are known to mediate simvastatin lactonization, simvastatin is administered as a lactone. Thus, the simvastatin lactone form detected in the blood is independent of UGT-mediated formation. Simvastatin is also metabolized in the intestine. In human intestinal microsomes, simvastatin lactone exhibited significant metabolism, with intrinsic clearance values corresponding to approximately 20 % of those observed in human liver microsomes [51]. The study by Deng et al. indicates that simvastatin is not transported by BCRP, MRP2, MRP3, MRP4, MRP8, or P-gp, which may be due to high passive permeability and may lead to false negative results in vesicle transport assays [52]. However, Li et al. discovered that up-regulation of P-gp expression leads to higher biliary excretion of simvastatin acid [93].

## 4 Drug interactions affect adverse reactions

### 4.1 Atorvastatin

Atorvastatin is metabolized by CYP3A4 and transported by proteins like OATP1B1/1B3, P-gp, or BCRP. When CYP3A4 or transporter inhibitors are combined with atorvastatin, the plasma levels of atorvastatin may significantly increase.

Significant increases in atorvastatin plasma levels were observed with the concurrent use of atorvastatin and cyclosporine, which inhibits CYP3A4 and OATP1B1. Concomitant administration of atorvastatin with several antiviral medications, azole antifungals, or macrolide antibiotics significantly increased atorvastatin plasma levels. This effect is primarily due to the inhibition of the CYP3A4 enzyme and transporters, which are involved in the metabolism and elimination of atorvastatin. Grapefruit juice has certain elements that block CYP3A4, potentially raising the plasma levels of drugs metabolized by CYP3A4. Grapefruit juice consumption can raise the plasma levels of atorvastatin. The aforementioned drugs or foods could increase the risk of atorvastatin-induced myopathy and rhabdomyolysis.

Fibrates (other than Gemfibrozil), Gemfibrozil may cause myopathy when given alone. Concomitant use of atorvastatin with fibrates or gemfibrozil increases the risk of myopathy and rhabdomyolysis. The concurrent use of atorvastatin with niacin at doses of 1 g or more per day or colchicine has been linked to cases of myopathy and rhabdomyolysis.

The simultaneous use of atorvastatin with rifampin, which acts as a CYP3A4 inducer and an inhibitor of OATP1B1, may result in unpredictable decreases in atorvastatin plasma levels [94].

### 4.2 Fluvastatin

Combination administration of fluvastatin with cyclosporine or fluconazole increases plasma exposure to fluatorvastatin. Fibrates and Gemfibrozil can lead to myopathy when used independently. Concomitant use of fluvastatin with fibrates or gemfibrozil increases the risk of myopathy and rhabdomyolysis. Additionally, the risk of skeletal muscle-related side effects may be heightened when fluvastatin sodium is combined with high doses of niacin (≥1 g/day) used for lipid modification. Fluvastatin, used in combination with colchicine, has been associated with cases of myopathy, including rhabdomyolysis [95].

### 4.3 Pravastatin

Pravastatin is a substrate for the transporter protein OATP1B1. OATP1B1 inhibitor administered with pravastatin concurrently can lead to a significant increase in the plasma concentration of pravastatin.

The co-administration of pravastatin sodium tablets with colchicine, fibrates (excluding gemfibrozil), gemfibrozil, niacin, or cyclosporine raises the risk of myopathy and rhabdomyolysis. Similarly, the concurrent use of clarithromycin or erythromycin with pravastatin sodium tablets increases the likelihood of myopathy and rhabdomyolysis. Other macrolide antibiotics, such as azithromycin, may also elevate pravastatin exposure and heighten the risk of myopathy and rhabdomyolysis when used together with pravastatin.

Additionally, the simultaneous use of pravastatin with bile acid sequestrants like cholestyramine or colestipol reduces the average exposure to pravastatin by approximately 51 % and 47 %, respectively [96].

### 4.4 Lovastatin

Lovastatin, similar to other HMG-CoA reductase inhibitors, is metabolized by CYP3A4. Medications that inhibit this enzyme can elevate lovastatin plasma concentrations, increasing myopathy risk. These drugs include itraconazole, ketoconazole, posaconazole, voriconazole, the macrolide antibiotics erythromycin and clarithromycin, the ketolide antibiotic telithromycin, HIV protease inhibitors, boceprevir, telaprevir, the antidepressant nefazodone, and products containing cobicistat. The combination of lovastatin with cyclosporine, gemfibrozil, other lipid-lowering drugs (such as other fibrates or niacin at doses ≥1 g/day), ranolazine, colchicine, or amiodarone increases the risk of myopathy and rhabdomyolysis [97]. Taking lovastatin with food can enhance its bioavailability.

### 4.5 Pitavastatin

Cyclosporine, erythromycin, and rifampin significantly increase the exposure to pitavastatin, thereby elevating the risk of myopathy and rhabdomyolysis. Gemfibrozil, as well as other fibrates, can cause myopathy when used alone. The risk of myopathy and rhabdomyolysis is further heightened when fibrates are used in combination with pitavastatin. Additionally, concurrent use of lipid-modifying doses of niacin (≥1 g/day) with pitavastatin may increase the risk of these adverse effects. Cases of myopathy and rhabdomyolysis have also been reported with the combined use of colchicine and pitavastatin [98].

### 4.6 Rosuvastatin

Rosuvastatin is metabolized by CYP2C9 and transported by transporters such as OATP1B1 and BCRP. Co-administration of CYP2C9 inhibitors and transporters can significantly increase the plasma concentration of rosuvastatin.

For example, cyclosporine raises rosuvastatin exposure by a factor of 7, while teriflunomide increases it by more than 2.5 times. Enasidenib, kamatinib, and fostamatinib each increase rosuvastatin exposure by more than 2 times, with enasidenib raising it by over 2.4 times and kamatinib by over 2.1 times. Febuxostat increases rosuvastatin levels by more than 1.9 times. Combining rosuvastatin with any of these drugs can increase the risk of myopathy and rhabdomyolysis.

Gemfibrozil significantly boosts rosuvastatin exposure, and taking gemfibrozil alone can lead to myopathy. The risk of myopathy and rhabdomyolysis is further elevated when gemfibrozil is used with rosuvastatin. Similarly, fibrates, in general, can cause myopathy, and their use in combination with rosuvastatin increases the risk of these muscle-related adverse effects. The concurrent use of niacin at doses ≥1 g/day with rosuvastatin has also been associated with an increased risk of myopathy and rhabdomyolysis.

Specific protease inhibitors can affect rosuvastatin exposure differently, which may also elevate the risk of myopathy. Specifically, simeprevir, a hepatitis C virus (HCV) protease inhibitor, and atazanavir/ritonavir or lopinavir/ritonavir (HIV-1 protease inhibitors) increase rosuvastatin exposure.

On the other hand, the concurrent use of aluminum hydroxide, magnesium hydroxide, or other antacids can reduce rosuvastatin exposure by about 50 % [96].

### 4.7 Simvastatin

Simvastatin is metabolized by the enzyme CYP3A4 and transported by the protein OATP1B1. Simvastatin exposure can significantly increase with the combined administration of CYP3A4 and OATP1B1 inhibitors.

Potent inhibitors of CYP3A4 include azole antifungals (such as itraconazole, ketoconazole, posaconazole, and voriconazole), macrolide antibiotics (like erythromycin and clarithromycin), HIV protease inhibitors (including nelfinavir, ritonavir, and darunavir/ritonavir), HCV protease inhibitors (such as boceprevir and telaprevir), cobicistat-containing products, and nefazodone. The risk of myopathy and rhabdomyolysis is heightened when simvastatin is taken alongside cyclosporine, danazol, gemfibrozil, other fibrates (except gemfibrozil), daptomycin, colchicine, niacin, amiodarone, dronedarone, ranolazine, or calcium channel blockers. Additionally, simvastatin exposure can be approximately doubled when taken with lomitapide, further increasing the risk of myopathy and rhabdomyolysis. Grapefruit juice has also been shown to elevate simvastatin plasma levels, potentially increasing the risk of these adverse effects [99].

## 5 Adverse effects of statins

Adverse effects of statins, although not common, have been reported, mainly including muscle toxicity, liver toxicity, gastrointestinal reactions, and some other symptoms. A retrospective study involving more than 100 000 patients on statin therapy in the U.S. found that 17.4 % stopped treatment because of statin-related adverse events. In order of frequency, the leading causes for discontinuation were myopathy (5075/107835; 4.7 %), musculoskeletal and connective tissue disorders not including myalgia or myopathy (2742/107835; 2.5 %), general symptoms (2493/107835; 2.3 %), liver injury (2308/107835; 2.1 %) [100]. Research conducted in China from 1989 to 2019 on adverse reactions to statin therapy revealed that gastrointestinal symptoms were the most frequent adverse reactions (1491/37828; 3.942 %), with liver disease following (486/37828; 1.285 %), muscle symptoms (444/37828; 1.174 %), and neurological symptoms (247/37828; 0.653 %) [101].

The incidence of gastrointestinal symptoms, myopathy, and liver disease varies for each statin. Fluvastatin (215/3094; 6.949 %) most commonly caused gastrointestinal adverse effects, followed by pravastatin (119/1988; 5.986 %), simvastatin (732/16 009; 4.572 %), lovastatin (138/3540; 3.898 %), and atorvastatin (260/11351; 2.291 %). The least likely to cause gastrointestinal irritation is rosuvastatin (27/1846; 1.463 %). The most common cause of liver injury was pravastatin (43/1988; 2.163 %), followed by fluvastatin (66/3094; 2.133 %), atorvastatin (148/11351; 1.304 %), simvastatin (200/16 009; 1.249 %), lovastatin (22/3540; 0.621 %) and rosuvastatin (7/1846; 0.379 %). There was no significant difference in muscle-related adverse reactions among statins, atorvastatin (190/11 351; 1.674 %), pravastatin (25/1988; 1.258 %), simvastatin (165/16 009; 1.031 %), rosuvastatin (17/1846; 0.921 %), lovastatin (26/3540; 0.734 %), and fluvastatin (21/3094; 0.679 %) [101].

The gastrointestinal reactions caused by statins are primarily mild, such as nausea, abdominal pain, diarrhea, bloating, etc.

Previous reports have shown that statins can treat osteoporosis. Still, recent studies have found that low-dose statin therapy is associated with a reduced risk of osteoporosis. In contrast, high-dose statin therapy is associated with an increased risk of osteoporosis [102]. Another clinical study also found that previous use of statins is associated with a reduced risk of osteoporosis in middle-aged women but is associated with an increased risk of osteoporosis in elderly women who have previously used statins [103]. However, the mechanism of high-dose statins affecting bone metabolism and leading to osteoporosis is still unclear. Statins may inhibit the body's cholesterol synthesis, decreasing serum hormone levels, which leads to osteoporosis [104,105].

Racial/ethnic factors may have a particular impact on the adverse effects caused by statins. The genetic background of statin-related myopathy in East Asians is different from that of Europeans and Americans [106]. Asian Americans may be sensitive to statin doses, while African Americans and Hispanic/Latino Americans are less sensitive to statin doses compared to non-Hispanic white Americans. Compared with white people, Japanese, Chinese, Malay, and Indian people have higher plasma levels of statin, so a lower initial dose should be used [107].

Compared to statins themselves, the metabolites of statins are more likely to cause adverse effects. Clinical pharmacokinetic studies by Hermann et al. showed that patients with atorvastatin-associated myopathy had no significant differences in systemic exposure to atorvastatin compared to healthy controls but had 2.4 times higher exposure to the metabolite atorvastatin lactone and 3.1 times higher exposure to hydroxy atorvastatin [108]. In a clinical trial involving 28 patients with coronary heart disease and subjective statin-associated myopathy, the ratio between atorvastatin lactone and acidic forms of atorvastatin metabolites was higher in the patients intolerant to statins [109]. The inhibitory effect of the lactone form of statins on the activity of MDR1, CYP2C9, CYP3A4, and CYP3A5 has been found to correlate to lipophilicity, and such inhibition affects drug metabolism, transportation, and interactions [110].

Ine et al. found that the lactone forms of atorvastatin, fluvastatin, pravastatin, and simvastatin were more toxic to primary human skeletal muscle cells than their acidic forms [111]. Based on undifferentiated and differentiated C2C12 cells, Taha et al. demonstrated that pH can influence the interconversion between lactone and hydroxy acid forms of simvastatin and pravastatin [112]. Under physiological and alkaline pH conditions, the lactone forms convert to acidic forms, potentially preventing statin-induced myotoxicity. Using primary hepatocytes from rainbow trout, Ellesat et al. showed that the lactone forms of atorvastatin and simvastatin were more toxic to liver cells [113].

Although statin-lactone metabolites do not inhibit HMGCR activity, their high lipophilicity may increase accumulation in hepatocytes or muscle cells through passive diffusion. Alternatively, these metabolites may be converted to active acidic forms, inhibiting protein isoprenylation and leading to hepatotoxicity and myotoxicity.

### 5.1 Statin-induced muscle toxicity

Myopathy is the predominant adverse effect of statins, accounting for approximately 95 % of the cases. About 10–15 % of individuals taking statins may experience muscle-related adverse effects during treatment [114]. These adverse events can manifest as muscle pain, myopathy, or myositis accompanied by elevated creatine kinase (CK) levels. In rare cases, patients may progress to the most severe, life-threatening condition known as rhabdomyolysis, characterized by serum CK levels exceeding 10 times the upper limit of normal, along with myoglobinuria, renal impairment, and electrolyte disturbances [115]. Apart from rhabdomyolysis, statins have also been linked to a rare condition known as statin-induced necrotizing autoimmune myopathy (SINAM), an autoimmune myopathy confirmed by the presence of autoantibodies specific to HMGCR [116].

In observational studies, around 10 % of people experience muscle pain without increased muscle enzymes, but this rate is significantly higher in clinical practice. Research from 21 clinical trials of statins indicated that myopathy appears in 5 patients for every 100 000 person-years. Rhabdomyolysis occurs infrequently, with a rate of 1 patient in 100 000 people. In Canada and the United States, analysis of statin-related rhabdomyolysis case reports indicates an average of 824 cases each year, with a dose-response relationship evident at increased statin doses [117]. Autoimmune myopathy is a rare disorder seen in only 9 to 14 out of 100 000 people, with roughly 10 % of patients having anti-SRP or anti-HMGCR myopathy. Statin exposure among HMGCR-resistant myopathy patients varies by cohort source, with US cohorts reporting 38 %–63 % exposure and 44 % in European cohorts [118].

**Fig. 3:**
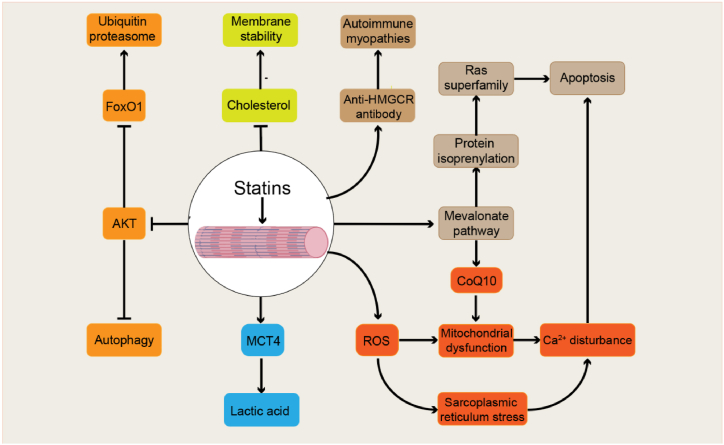
**Possible mechanisms of statin-induced muscle injury.** The possible mechanisms of muscle damage caused by statins include lowering cholesterol to affect membrane stability, reducing protein isoprenylation to affect cell function, affecting lactate transporters to cause lactate accumulation in muscle cells, affecting the AKT signaling pathway to cause autophagy, activating the ubiquitin-proteasome system to cause protein degradation, inducing mitochondrial and sarcoplasmic reticulum dysfunction through oxidative stress and calcium ion imbalance to induce cell apoptosis, and immune-mediated anti HMGCR myopathy.

Different types of muscle fibers exhibit varying resistance to statin-induced muscle damage. Oxidative-type fibers rich in mitochondria (Type I fibers) resist statin-induced myopathy, whereas glycolytic fibers (Type II fibers) are more susceptible to injury [119]. Singh et al. found that atorvastatin selectively impairs mitochondrial function in glycolytic muscle fibers and induces a shift from oxidative IIA to glycolytic IIB fibers [120]. This phenomenon is related to the expression of peroxisome proliferator-activated receptor gamma coactivator 1β (PGC-1β) in skeletal muscle, which effectively defends against statin-induced muscle damage, and PGC-1α similarly possesses this protective effect. Statins also impair the expression of PGC-1β in human and rodent skeletal muscles [120,121]. The exact mechanisms underlying statin-induced myopathy remain unclear, even with several theories proposed (Fig. 3). These theories include the effect of statins on lowering cholesterol levels in muscle cell membranes and reducing intermediate products in the mevalonate pathway, such as isopentadiene and electron transport chain proteins, which affect cell function and cause apoptosis. In addition, statins also affect the AKT signaling pathway, lactate uptake, protein degradation, and cellular Ca^2+^ concentration.

#### 5.1.1 Membrane stability

As a significant lipid constituent of cell membranes, cholesterol is crucial for maintaining membrane fluidity and permeability. Cholesterol also regulates key membrane protein activity in mammalian cells, which regulates protein activity by binding to specific sites on membrane proteins [122]. Therefore, statins inhibit HMGCR-controlled cholesterol synthesis, and cholesterol depletion in muscle cell membranes may affect membrane stability and trigger myopathy [123].

#### 5.1.2 Decreased protein isoprenylation affects cell function

In addition to cholesterol, statins reduce the production of other important metabolites, including coenzyme Q10 and other isoprenoid proteins necessary for normal skeletal muscle cell function. Protein isoprenylation is a post-translational modification where a 15-carbon farnesyl or a 20-carbon geranylgeranyl isoprenoid moiety is added to specific cysteine residues near the C-terminus of target proteins. This modification process is necessary for the biosynthesis of cholesterol intermediates, FPP and GGPP. Enzymes catalyzing protein isoprenylation include farnesyltransferase (FTase) and geranylgeranyltransferase (GGTase). There are two forms of GGTase, GGTase-I and GGTase-II, each with different substrate specificities. Prenylated proteins ultimately localize to the plasma or intracellular membranes, serving various critical protein functions [124].

Many important proteins are substrates for protein isoprenylation, especially the small GTPase Ras superfamily proteins such as Ras, Rho, Rab, Ran, and Arf, as well as nuclear lamins. The Ras superfamily of small GTPases act as molecular switches involved in regulating diverse cellular activities including cytoskeletal reorganization, gene expression, cell wall synthesis, cell cycle progression, vesicle trafficking, nucleocytoplasmic transport, microtubule formation, and MAP kinase signaling transduction [125] Therefore, statin-induced reduction in the synthesis of FPP and GGPP, crucial for the isoprenylation of key proteins, is a potential mechanism contributing to statin-induced myopathy [126].

Mullen et al. demonstrated that simvastatin reduced *de novo* cholesterol synthesis in human C2C12 myoblasts and hepatocyte cell line HepG2 [127]. Concurrently, levels of Ras and Rap1 isoprenylation were also decreased in both cell lines, along with reduced N-linked glycosylation, specifically in C2C12 myoblasts. However, cytotoxicity was only observed in C2C12 myoblasts. In another *in vitro* study, lovastatin and pravastatin were found to inhibit geranylgeranylation of low molecular weight proteins in cultured rat myoblasts, which suppressed protein synthesis and disrupted myotube morphology after prolonged exposure [128]. A supplementation with farnesol and geranylgeraniol significantly ameliorated the morphological changes induced by HMGCR inhibitors in muscle cells [129]. Moreover, using rat L-6 myoblast differentiation into myotube tissue, Johnson et al. found that adding mevalonic acid and geranylgeraniol (GG-OH) prevented statin-induced apoptosis, while farnesol, however, had partial effects [130].

#### 5.1.3 Lactic acid buildup in muscle cells

Accumulation of lactate in muscle cells is a major cause of muscle soreness. Monocarboxylate transporters (MCTs) 1 and 4 are the primary lactate transporters in skeletal muscle. MCT1 is responsible for lactate transport in glycolytic type II fibers, and MCT4 handles lactate transport in oxidative type I fibers. Kikutani et al. found that atorvastatin reduced cell viability in a dose-dependent manner and upregulated mRNA levels of MCT4 but not MCT1. Knockdown of MCT4 countered the reduction of cell viability and apoptosis induced by atorvastatin, simvastatin, and fluvastatin [131]. This discovery suggests that statin-induced cytotoxicity is related to lactate transport. The differential regulation of MCT1 and MCT4 expression by statins also explains the muscle-type specificity of statin-induced muscle damage.

#### 5.1.4 AKT signaling pathway and the ubiquitin-proteasome system

The AKT signaling pathway activates the mammalian target of rapamycin (mTOR) to regulate protein synthesis, which is crucial for muscle growth [132]. The PI3K/AKT/mTOR signaling pathway also plays a significant role in autophagy regulation, directly influencing cell survival [133]. The previous experimental results showed that statins impair AKT activation in C2C12 myotubes and mouse skeletal muscle, leading to muscle atrophy and apoptosis, with lipophilic statins exhibiting higher toxicity than hydrophilic statins [134]. Sanvee et al. found that simvastatin inhibited mTORC2 in C2C12 cells and mice, impairing Akt Ser473 phosphorylation and exhibiting cytotoxicity in C2C12 myoblasts. This process is primarily due to simvastatin inhibiting Rap1 protein isoprenylation, leading to mitochondrial dysfunction and reactive oxygen species (ROS) accumulation [135].

The ubiquitin-proteasome system is the principal pathway for intracellular protein degradation. The AKT signaling pathway negatively regulates forkhead box O (FoxO) to inhibit the upregulation of several ubiquitin-proteasome and autophagy-related genes. Hanai et al. discovered that statins induce muscle-specific ubiquitin ligase atrogin-1/MAFbx pathways, activating the ubiquitin-proteasome pathway, which enhances muscle protein degradation and induces muscle toxicity [136].

#### 5.1.5 Mitochondrial dysfunction

Mitochondria are the primary sites for aerobic metabolism in skeletal muscle cells, which consume high energy and contain many mitochondria. The tricarboxylic acid (TCA) cycle generates NADH, which oxidizes the mitochondrial electron transport chain to produce ATP. This process requires O_2_, coenzyme Q10 (CoQ10), and cytochrome *c*. Besides energy production, mitochondria are also involved in apoptosis-related pathways. Mitochondrial dysfunction is the primary cause of muscle damage induced by statins. Statins affect mitochondrial cofactor CoQ10 formation via the mevalonate pathway, leading to mitochondrial dysfunction and subsequently disrupting cellular respiration, resulting in muscle damage [137]. However, the *in vitro* results suggest that statin-induced apoptosis is unrelated to ubiquinone depletion [130]. 9 previous clinical trials assessed the effect of CoQ10 supplementation on statin-induced muscle symptoms, with 5 showing beneficial effects and the other 4 showing no effect [[138], [139], [140], [141], [142], [143], [144], [145], [146]].

Oxidative stress is also a significant factor in statin-induced mitochondrial dysfunction and muscle damage. Statins can increase mitochondrial H_2_O_2_ production, alter Bax/Bcl-2 ratios, and induce DNA damage in muscle biopsies of statin-associated myopathy patients. Additionally, muscle phenotype may influence statin-induced muscle symptoms, with oxidative muscle fibers showing more excellent resistance to statin-related toxicity than glycolytic fibers [119]. Bouitbir et al. found that statin-treated patients exhibit reduced ROS production and enhanced oxidative capacity, with widespread mRNA upregulation of the PGC-1 family. However, in muscle biopsies of statin-induced myopathy patients, oxidative capacity decreases, ROS increases, and PGC-1 mRNA expression collapses. ROS production triggers mitochondrial biogenesis pathways and enhances cardiac antioxidant capacity in atorvastatin-induced scenarios. Conversely, increased ROS production post-treatment in skeletal muscle induces mitochondrial damage and reduces mitochondrial biogenesis mechanisms [147].

Additionally, Schirris et al. found that statin lactones specifically inhibit the enzyme activity of respiratory chain Complex III, reducing Complex III activity in statin-induced myopathy patients [148]. However, simvastatin-induced mitochondrial damage was reported to be caused by inhibiting respiratory chain Complex I in human skeletal muscle [149].

#### 5.1.6 Calcium ion imbalance

Calcium ions are vital regulators and signaling molecules in muscle fibers, essential for controlling muscle function and activity [150]. Mitochondrial dysfunction can also disrupt intracellular Ca^2+^ balance. Statins impact sarcoplasmic reticulum and mitochondrial Ca^2+^ release, thereby influencing calcium homeostasis and increasing cytoplasmic calcium ion concentration [151]. A sudden rise in cytoplasmic calcium ion concentration can lead to the generation of free radicals and disruption of function across various organelles [152]. Additionally, elevated Ca^2+^ activates intracellular calcium protease Calpain, which, through Caspase-mediated cascades, ultimately induces cell apoptosis [153]. In addition, Ca^2+^ is also involved in activating DNA nucleases, leading to the degradation of nuclear DNA [154].

#### 5.1.7 Immune-mediated anti-HMGCR myopathy

The pathogenesis of immune-mediated anti-HMGCR myopathy currently lacks definitive conclusions. It is hypothesized that statin exposure may lead to overexpression of HMGCR in various tissues and cell types, initiating autoimmune responses and resulting in sustained autoimmune reactions through positive feedback loops, ultimately leading to muscle fiber atrophy and degeneration [155].

### 5.2 Hepatotoxicity induced by statins

**Fig. 4:**
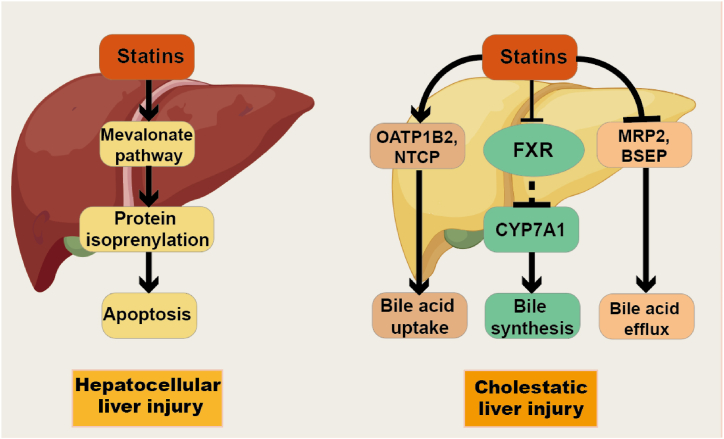
**Statin-induced liver injury.** Statins can induce hepatocellular and cholestatic toxicity. Hepatocellular liver injury is similar to muscle injury, as the inhibition of protein isoprenylation by statins can cause mitochondrial damage, leading to cell apoptosis. Cholestasis-induced liver injury is a combination of three aspects: statins inhibit FXR to promote CYP7A1 expression and induce hepatic bile synthesis, statins upregulate OATP1B2 and NTCP expression to enhance bile reabsorption, statins competitively inhibit efflux proteins BSEP and MRP2 and reduce the expression of efflux proteins BSEP, OSTα and OSTβ to inhibit bile efflux.

Liver toxicity from statins primarily presents as elevated serum transaminases and cholestasis following long-term use (Fig. 4). Hepatocellular pattern hepatotoxicity is defined by a predominant rise in alanine aminotransferase (ALT) [156]. Most patients experience liver injury within three months of starting treatment. Statin therapy is associated with elevated liver transaminases in up to 1–3% of patients [157], often in a dose-dependent manner and more likely in cases of high-dose statin use, concomitant use with other lipid-lowering agents like fibrates, drugs sharing metabolic pathways with statins, or in elderly patients or those with severe hepatic or renal impairment [158]. In most cases, transaminase elevations resolve or diminish upon discontinuation of treatment. Cholestasis is also a common type of liver injury associated with statin use; analysis of suspected adverse drug reactions reported to the Swedish Medical Products Agency from 1988 to 2010 indicated that 34 % of statin-induced liver injury patients developed jaundice. Atorvastatin and simvastatin are the statins most commonly implicated in drug-induced liver injury, with simvastatin primarily associated with hepatocellular damage and atorvastatin with cholestatic liver injury [159]. Up to now, different hepatotoxic mechanisms of statins have been proposed. However, most of the hepatotoxic mechanisms have not been fully elucidated.

#### 5.2.1 Hepatocellular pattern hepatotoxicity

The reasons for statin-induced hepatocellular pattern hepatotoxicity are similar to those of statin-induced muscle cells, involving competitive inhibition of HMGCR that reduces the synthesis of mevalonic acid and its downstream products, FPP and GGPP. The regulation affects protein isoprenylation, impacting small GTPase Ras superfamily signaling pathways or mitochondrial function. Mitochondria, as the energy factory of cells, participate in multiple critical physiological processes such as cellular metabolism, signal transduction, differentiation, growth, apoptosis, and death. When mitochondria are damaged, they can release pro-apoptotic factors to cause cell apoptosis [160]. In HepG2 cells, simvastatin was shown by Tavintharan et al. to decrease coenzyme Q10 levels in mitochondria, induce cell death, increase DNA oxidative damage, and reduce ATP synthesis [161]. *In vitro* experiments have been used to evaluate statin-induced mitochondrial damage in HepG2 cells from multiple perspectives, including mitochondrial superoxide production, mitochondrial membrane potential, mitochondrial permeability transition, intracellular calcium concentration, and apoptotic cell count. The results indicated that simvastatin, lovastatin, fluvastatin, atorvastatin, pravastatin, and rosuvastatin caused mitochondrial damage in HepG2 cells [162].

#### 5.2.2 Cholestasis

As the key components of bile, bile acids are produced from cholesterol in the liver through the classical and alternative pathways, creating free primary bile acids. These primary bile acids are combined with taurine, glycine, or sulfate and glucuronic acid to form conjugated bile acids, which are then secreted into the bile ducts to participate in the enterohepatic circulation [163]. Cholestasis occurs when this normal bile acid cycle is disrupted, leading to excessive accumulation of bile acids and other harmful substances in the liver, resulting in liver damage [164]. Statins primarily cause cholestasis by increasing the synthesis and uptake of bile acids in the liver. Fu et al. found that atorvastatin induced CYP7A1 expression and enhanced bile acid synthesis by inhibiting FXR signaling in the liver and intestines [165]. Byun et al. demonstrated that pravastatin induces CYP7A1 expression, thereby increasing bile acid synthesis [166]. Additionally, atorvastatin increases the expression of the unconjugated bile acid uptake transporter OATP1B2 and the conjugated bile acid uptake transporter NTCP by enhancing bile acid uptake [165]. Li et al. also observed similar effects in ApoE−/− mice fed a high-fat diet [167], suggesting that statins promote bile acid synthesis and reabsorption by upregulating CYP7A1, OATP1B2, and NTCP.

On the other hand, statins affect bile excretion, and drugs like atorvastatin and pravastatin inhibit the bile acid efflux transporters BSEP and MRP2 [67,81], inhibiting bile acid elimination out of hepatocytes. Statins may competitively bind to the bile acid efflux transporters to reduce their efficiency in excreting bile acids. Hirano et al. found competitive inhibition between taurine-conjugated bile acids (TCA) and pravastatin, indicating that statins inhibit human and rodent BSEP-mediated TCA efflux [67]. Furthermore, Li et al. demonstrated that atorvastatin reduced the expression of bile acid transporters BSEP, OSTα, and OSTβ [168], interrupting bile acid transportation.

## 6 Genetic polymorphisms of statin-metabolizing enzymes and transporters

Genetic polymorphisms of metabolizing enzymes and transporter genes play a significant role in the variation of therapeutic and adverse effects of statins [9]. Adverse reactions to statins are often dose-dependent, with the incidence increasing as plasma concentrations in the blood rise. This phenomenon is primarily due to genetic defects in statin-metabolizing enzymes and transporters. SLCO1B, also known as OATP1B, is a transporter in the liver responsible for uptaking various endogenous and exogenous compounds from the portal vein blood. Clinical studies have shown that polymorphisms in SLCO1B1 significantly impact the pharmacokinetics of multiple statins and their metabolites, as well as their drug interactions and adverse effects [70,169,170]. After taking rosuvastatin, plasma rosuvastatin and its metabolites are notably higher in Chinese, Malay, and Asian Indian populations than in Caucasians due to SLCO1B1 gene polymorphisms [171]. Multiple studies demonstrate that polymorphisms in efflux proteins ABCB1, ABCC2, and ABCG2 genes significantly impact the pharmacokinetics of statins and their metabolites, as well as the lipid-lowering and adverse effects of statins [[172], [173], [174], [175], [176], [177], [178], [179], [180]]. Loss of functional polymorphisms in SLCO1B1 and ABCG2 genes is linked to myotoxicity and hepatotoxicity associated with rosuvastatin [181].

Polymorphisms in phase I CYP450 enzymes also affect the pharmacokinetics of statins and their metabolites [[182], [183], [184]]. CYP3A gene polymorphisms are associated with muscle injury induced by atorvastatin [184]. The pharmacokinetics of statins are also influenced by phase II UGT enzyme polymorphisms, which affect the lactonization of the drug [185,186]. UGT1A3∗2 polymorphism is associated with increased lactonization of atorvastatin and may influence its lipid-lowering effects [187]. Increased levels of statin metabolites in the body due to the polymorphisms of statin metabolizing enzymes and transport proteins may be one of the reasons for statin-induced hepatotoxicity and myotoxicity.

## 7 Regulation of statin metabolism and transportation

Currently, there are no targeted treatment methods for adverse effects caused by statins. Generally, discontinuation of statin therapy is chosen when toxic events occur. After discontinuation and resolution of symptoms, options include intermittent administration at lower doses or switching to alternative lipid-lowering agents such as ezetimibe, bile acid sequestrants, or fibrates, which have a lower risk of inducing adverse effects compared to the original statin therapy [188]. Concurrently, some studies suggest supplementing CoQ10 and vitamin D for patients experiencing statin-induced muscle injury [[189], [190], [191]], but clinical studies did not support this proposal [138,192]. Therefore, it is crucial to study further how to enhance patient tolerance to statins and reduce adverse reactions.

Due to the significant impact of metabolic processes on both adverse effects and the therapeutic efficacy of statins, regulating statin metabolism is considered pivotal. Targeted regulation of transporters and metabolic enzyme expression by transcription factors proves effective in enhancing statin therapy or preventing toxic accidents. Nuclear receptors (NRs) are critical intracellular receptor proteins that sense signals from in or outside cells, inducing target genes' transcriptional responses upon ligand binding. Nuclear receptors such as pregnane X receptor (PXR) and constitutive androstane receptor (CAR) are predominantly expressed in the liver and gastrointestinal tract, acting as the two most essential sensors for endogenous and exogenous stimuli crucial for detoxification through regulation of phase I and II metabolic enzymes and phase III transporters [193]. Transcription factors involved in the regulation of statin metabolism and transportation are summarized in Table 2.

**Table 2:** Transcription factors regulate statin metabolism and transportation.

	Phase I Metabolic Enzyme	Phase Ⅱ Metabolic Enzyme	Uptake Protein	Efflux Protein
PXR	CYP3A4, CYP2B6, CYP2c8, CYP2c9, CYP2c19	UGT1A1, UGT1A3, UGT1A4, SULT2A1	OATP1A2, OATP2	P-gp, MRP2, MRP3
CAR	CYP2B, CYP3A4, CYP2C8, CYP2C9, CYP2C19	UGT1A1, SULT2A1	OATP1B3, OATP2B1	P-gp, MRP2, MRP4
PPAR α		SULT2A1, UGT1A1, UGT1A3, UGT1A4, UGT1A6, UGT2B4		
AHR	CYP1A1, CYP1A2, CYP1B1	UGT1A1, UGT1A3, UGT1A4, UGT1A6	NTCP, OATP1B1,	BCRP
KLF15	CYP2B9, CYP3A4, CYP2E1	UGT1A9, SULT1A1, SULT2A1,	NTCP, OATP1A1	BSEP, MDR1, MRP2

PXR can be activated by various chemicals, such as glucocorticoids, macrocyclic antibiotics, antifungal drugs, herbal extracts, and even natural products from the human gut microbiota. None of these share apparent structural similarities [194]. Both acidic and lactone forms of atorvastatin and its hydroxylated metabolites serve as ligands for PXR with varying activating effectiveness, although hydroxylated metabolites show weaker induction [195].

PXR is widely recognized as a key transcriptional regulator that induces CYP3A4 expression [196]. PXR also regulates other phase I enzymes including CYP2B6, CYP2C8, CYP2C9, CYP2C19 [[197], [198], [199], [200]], phase II enzymes UGT1A1, UGT1A3, UGT1A4, SULT2A1 [[201], [202], [203]], uptake transporters OATP1A2, OATP2 [204,205] and efflux transporters P-gp, MRP2, MRP3 [[206], [207], [208]]. Thus, regulating PXR can influence the entire metabolism and disposition of statins. In addition, activation of PXR suppresses CYP7A1 gene expression and bile acid synthesis, thereby reducing bile accumulation-induced liver damage [209]. Suppressing PXR enhanced hepatic toxicity of atorvastatin in primary rat cells and female Sprague-Dawley rats [210], which might result from reduction of CYP3A4, P-gp, and OATP1B1 transporters due to PXR inhibition, slowing the statin metabolism and causing the drug overdose in blood circulation [7]. Therefore, activating PXR may enhance the liver's uptake, metabolism, and transportation of statins. These processes could speed up drug disposition, lower blood statin levels, decrease statin and metabolite uptake in muscle cells, and reduce the risk of muscle-related adverse effects. The activation of PXR can also help reduce bile acid accumulation-leading liver damage by inhibiting bile acid synthesis and improving the metabolism and transportation of statins. Long-term administration of rifampicin caused lower plasma concentrations of simvastatin, atorvastatin, and their metabolites [211,212], as rifampicin acts as a specific agonist to activate human PXR, inducing statin-metabolizing enzymes and efflux transporters. However, reducing statins in the blood may affect the cholesterol-lowering efficacy of the drugs. Meanwhile, short-term administration with rifampicin competitively inhibits the uptake of statins by OATP1B in the liver, increasing the circulatory levels of the drugs [213]. However, rosuvastatin is not a substrate of CYP enzymes, so the impact of rifampicin as a PXR ligand on its pharmacokinetics is insignificant [214].

CAR heterodimerizes with retinoid X receptor (RXR) to constitutively activate genes containing CAR response elements in the absence of ligands. Hence, CAR was early designated as a constitutively active nuclear receptor [215]. Studies have suggested that certain statins such as simvastatin, lovastatin, fluvastatin, and atorvastatin act as agonists for human, rat, and mouse CAR [216], although subsequent studies questioned the activation by statins, noting that atorvastatin is not an agonist that unbinds to human CAR [195]. Under stimulation by phenobarbital (PB) as a known agonist of CAR, CAR/RXR heterodimers translocate into the nucleus, transactivating a distal enhancer termed the phenobarbital-responsive enhancer module (PBREM), thereby inducing transcription of CYP2B [217]. CAR also regulates OATP1B1, OATP2B1, CYP3A4, CYP2C8, CYP2C9, CYP2C19, UGT1A1, SULT2A1, P-gp, MRP2, and MRP4 [[218], [219], [220]], majority of which are critical for the metabolism and transportation of statins.

Notably, many drugs that induce expressions of CYP enzymes via CAR and PXR may significantly reduce the bioavailability of statins, thus decreasing the therapeutic efficacy of statins [221]. Despite sharing many common gene targets, PXR and CAR play similar roles in regulating the metabolism of statins. However, recent studies indicate that PXR and CAR can form heterodimers that mutually inhibit each other [222], suggesting that targeting the regulation of PXR or CAR requires considering their interaction.

## 8 Discussion

Precision medicine (PM) helps determine the best disease prevention or treatment by considering individual genetic backgrounds, environments, and lifestyles. The absorption, uptake, metabolism, and excretion of statins significantly influence their therapeutic and adverse effects. Genetic polymorphisms can alter the blood concentration of statins and their metabolites, leading to individual differences in clinical use. Pharmacogenetics and pharmacogenomics studies can help us better understand inter-individual differences, enabling the development of personalized statin therapy plans using big data and machine learning [223].

Due to the uncertainty in patients' conditions and genetic backgrounds, there is sometimes a need for high drug doses while minimizing toxic adverse effects as much as possible. Transcriptional regulation of metabolism becomes particularly important in such cases. The transport and metabolism of statins are complex processes involving numerous enzymes and transporters, making it challenging to regulate them effectively by targeting a single enzyme or transporter. Activating nuclear receptors through corresponding agonists or delivering transcription factors mRNA or siRNA via nanomaterials may offer a more efficient solution. PXR and CAR are binding to a wide range of structurally diverse ligands, which makes them highly valuable for research, whether using existing PXR and CAR agonists or antagonists or designing and improving drugs based on existing structures. Studies have shown that using a PXR agonist like rifampicin can alter the plasma exposure of atorvastatin, potentially affecting its therapeutic efficacy and adverse effects [224].

In addition to PXR and CAR, other transcription factors may also regulate the homeostatic metabolism of statins, although no literature currently supports this. However, we can envision their potential role in the regulation of statin metabolism. Peroxisome proliferator-activated receptors (PPARs) are a class of ligand-inducible nuclear receptors that, through heterodimerization with RXR and binding to peroxisome proliferator response elements (PPREs) in target gene promoters, integrate the expression of a wide range of genes involved in cellular lipid metabolism, energy homeostasis, and inflammation. Among the three subtypes PPARα (NR1C1), PPARδ/β (NR1C2), and PPARγ (NR1C3) [225], PPARα plays a crucial role in the transcriptional regulation of phase II enzymes. PPARα induces the expression of human liver SULT2A1 [226], and several human liver UGTs, including UGT1A1, UGT1A3, UGT1A4, UGT1A6, and UGT2B4, have shown transcriptional induction in response to PPARα activation [227,228]. Given the crucial role of phase II enzyme UGTs in converting statins into inactive and highly lipophilic lactone forms, PPARα regulation of UGTs is also a possible target for enhancing statin efficacy and reducing adverse effects. However, PPARα is crucial for promoting fatty acid oxidation and maintaining energy homeostasis, and therefore indiscriminately inhibiting PPARα activity potently promotes obesity and non-alcoholic fatty liver disease (NAFLD), especially among patients with hyperlipidemia who are already suitable for statin therapy [229].

The aryl hydrocarbon receptor (AHR), a basic helix-loop-helix Per-Arnt-Sim (bHLH-PAS) superfamily member, is an evolutionarily conserved ligand-activated transcription factor with functions similar to nuclear receptors. AHR can modulate cellular responses to xenobiotics by upregulating the expression of drug-metabolizing enzymes and transport proteins [230]. Upon activation, AHR enhances the expression of target genes such as CYP450 enzymes CYP1A1, CYP1A2, and CYP1B1, as well as several members of the UGT1A family (UGT1A1, UGT1A3/1A4, UGT1A6) and drug transport proteins including SLC10A1/NTCP, SLCO1B1/OATP1B1, and ABCG2 [219,231,232]. Targeted regulation of AHR may modulate drug transporters of statins, affecting drug therapy and adverse reactions. Notably, activated AHR can induce CAR expression without affecting PXR, which could also impact statin metabolism [233]. Additionally, hepatic AHR activation induces CD36 gene expression and increases fatty acid uptake, promoting hepatic steatosis [234]. The activation also contributes to dyslipidemia and atherosclerosis induced by a high-fat diet [235]. However, research suggests that AHR can coordinately downregulate cholesterol biosynthesis genes in a DRE-independent manner [236]. Therefore, the AHR-driven regulations above are potentially associated with statins' therapeutic and adverse effects.

Kruppel-like factors (KLFs) are essential transcription factors in eukaryotes that are highly conserved at the carboxyl-terminal end possessing three tandem zinc finger motifs. Sen-Banerjee found that mevastatin, simvastatin, and lovastatin can induce KLF2 expression, and reduced KLF2 expression attenuates statin-mediated accumulation of eNOS and platelet-derived growth factor levels [237]. KLF15, another family member, is expressed in metabolically active organs and tissues and regulates many metabolic processes [238]. Our previous studies revealed that hepatic KLF15 (in)directly and broadly regulates Phase I-III enzymes and transporters of several endogenous and exogenous substances, including bile acids, steroid hormones, acetaminophen, and rifampicin [37,239]. KLF15 exerts inhibitory effects on enzymes and transporters such as CYP2B9, CYP3A4, UGT1A9, SULT1A1, SULT2A1, MDR1, MRP2, and BSEP while inducing the expression of CYP2E1, NTCP, and OATP1A1. Liver-specific knockout of KLF15 increases the expression levels of detoxifying enzymes and transporters for acetaminophen and rifampicin while reducing enzymes that exacerbate toxicity, thereby mitigating liver toxicity induced by drugs [37,239]. Targeted regulation of KLF15 may optimize the metabolism-related systems of statin drugs, effectively countering statin-induced liver toxicity and muscle damage. However, the high potency requires deep investigation into the KLF15-based regulation and underlying mechanisms of statin metabolism.

Significant interindividual variability in the pharmacokinetics and pharmacodynamics of statins results from the genetic expression of genes involved in their absorption, distribution, metabolism, and excretion, which genetic polymorphisms cannot solely explain. Epigenetic factors also play crucial regulatory roles, including DNA methylation, histone modifications, and transcriptional regulation mediated by non-coding RNAs (ncRNAs). Many genes encoding metabolic enzymes, transport proteins, transcription factors, and drug targets are under epigenetic control, exerting essential influences on the pharmacokinetics and pharmacodynamics of statins [240]. DNA methylation typically associates with gene transcription repression, altering chromatin structure to affect the binding of transcription factors or coactivators to target sites, thereby reducing gene expression. Histone modifications primarily consist of methylation (M), phosphorylation (P), and acetylation (A). The protein modifications can inhibit or activate transcription depending on the location and state.

For phase I metabolic enzymes, the dynamic methylation of cytosine-phosphate-guanine (CpG) sites is correlated with the expression of the CYP3A4 gene in adult liver, alongside highly variable CpG methylation sites within crucial CYP3A4 transcription factor binding regions [241]. Increased expression of Cyp3a16 in newborn mouse liver and Cyp3a11 in adult mouse liver correlates with increased H3K4me2, suggesting developmental transitions in gene expression linked to dynamic histone modifications during liver maturation post-birth [242]. Further, Oda discovered that low methylation in CpG-rich regions near the UGT1A1 promoter, coupled with high acetylation of histone H3 near the promoter, promotes liver-specific expression of UGT1A1 compared to the kidneys [243]. Yasar also found that differential methylation of CpG sites within known USF response elements, particularly at −4 CpG sites, explains individual variability in UGT1A1 expression and activity in the liver [244]. In Asian populations, Oeser observed that methylation regulates mRNA expression of liver UGT2B15, while low mRNA levels of UGT2B17 are due to common gene deletions rather than epigenetic causes [245]. Nie et al. noted dynamic changes in histone methylation, such as significant enrichment of H3K4me2 in adult liver and H3K27me3 in fetal liver, associated with developmental UGT1A1 expression [246]. Finally, methylation of ABCB1 DNA CpG sites influences the efflux of drugs like tacrolimus by regulating ABCB1 expression. Treatment with the methylation inhibitor 5-Aza-2-DC significantly reduces intracellular tacrolimus concentration [247]. CpGs associated with OATP1B2, NTCP, BSEP, and ABCG5/8 exhibit low liver methylation and high kidney and brain methylation. Additionally, histone H3 acetylation is high in the liver but minimal in the kidneys and brain, consistent with the tissue-specific distribution of these transport proteins [248].

MiRNAs, as the post-transcriptional regulators, also play crucial roles in cellular growth, proliferation, differentiation, and apoptosis. The expressions of phase I, II, and III metabolic enzymes and transporters are also under the regulation of miRNAs. For instance, Wei et al. found that has-miR-577, has-miR-1, hsa-miR-532-3p, and has-miR-627 significantly downregulate the translation efficiency of hepatic CYP3A4 mRNA [249]. MiR-122-5p and miR-378a-5p upregulate translational repression associated with CYP1A2, CYP3A4, and CYP2E1 [250]. MiR-491-3p regulates UGT1A1/UGT1A3/UGT1A6 [251], and miR-let7a-5p is involved in regulating ABCC2 expression [252]. Regulating the expression of transcription factors poses challenges. Adeno-associated viruses (AAV) are appealing vectors for gene therapy because of their unique life cycle, ability to infect non-dividing cells, and sustained expression in dividing cells with low pathogenicity. Using AAV vectors to target transcription factors is common, but it faces challenges such as cost, unstable quality control, and drawbacks like immune responses and delayed efficacy [253]. In contrast, miRNAs can be precisely regulated using nano-delivery systems to modulate the expression levels of specific metabolic enzymes or transport proteins, providing a more precise approach to regulation.

Statins are crucial for treating or preventing cardiovascular diseases and high cholesterol levels. This paper examines the metabolic processes, treatment mechanisms, adverse reactions, and correlations of statins. It also explores the potential regulation of statin metabolism for precise and personalized dosing. The paper discusses relevant transcription factors and epigenetic regulations, aiming to identify targets for accurate statin treatment.

To achieve precise medical research on statins, a multidisciplinary approach is necessary. This includes contributions from molecular biology, pharmacokinetics, pharmacogenetics, pharmacogenomics, materials chemistry, artificial intelligence, and extensive data analysis beyond biomedical fields. The hope is that through a deeper understanding of drug metabolism, advancements in gene therapy drugs targeting metabolic processes will lead to genuinely personalized precision medicine for statins, demonstrating the strength of the research.

## CRediT authorship contribution statement

**Zhuangqi Shi:** Writing – review & editing, Writing – original draft. **Shuxin Han:** Writing – review & editing, Funding acquisition.

## Institutional review board statement

Not applicable.

## Data availability statement

Not applicable.

## Funding

This research was funded by the 10.13039/501100001809National Natural Science Foundation of China (Grant No.32171167, S.H.), Tianchi Talent Introduction Plan Innovative Leader of Xinjiang Ugyur Autonomous Region (Grant No.51052401403, S.H.).

## Declaration of competing interest

We confirm that we have no financial or personal connections with individuals or organizations that could improperly influence our work. Additionally, we declare that we have no professional or personal interests in any product, service, or company that could affect the position in the manuscript above.

## Abbreviations:

CVD: cardiovascular diseases
HMGCR: hydroxy-3-methylglutaryl-coenzyme A reductase
HMG-CoA: hydroxy-3-methylglutaryl-coenzyme A
LDL-C: low-density lipoprotein cholesterol
HDL-C: high-density lipoprotein cholesterol
EXM: endobiotic and xenobiotic metabolism
CYP: cytochrome P450
UGT: UDP-glucuronosyltransferases
SULT: sulfotransferases
GST: glutathione S-transferases
ABC: ATP-binding cassette
SLC: solute carrier
OATP: organic anion transporting polypeptide
OCT: organic cation transporter
OAT: organic anion transporter
P-gp: P-glycoprotein
BCRP: breast cancer resistance protein
MRP: multidrug resistance-associated protein
NTCP: Na^+^/Taurocholate co-transporting polypeptide
BSEP: bile salt export pump
T_max_: time to peak drug concentration
CK: creatine kinase
SINAM: statin-induced necrotizing autoimmune myopathy
FPP: farnesyl pyrophosphate
GGPP: geranylgeranyl pyrophosphate
FTase: farnesyltransferase
GGTase: geranylgeranyltransferase
GG-OH: geranylgeraniol
MCTs: monocarboxylate transporters
mTOR: the mammalian target of rapamycin
FoxO: forkhead box O
PGC-1: peroxisome proliferator-activated receptor gamma coactivator 1
ROS: reactive oxygen species
TCA: taurine-conjugated bile acids
NRs: nuclear receptors
PXR: pregnane X receptor
CAR: constitutive androstane receptor
RXR: retinoid X receptor
PPARs: peroxisome proliferator-activated receptors
NAFLD: non-alcoholic fatty liver disease
AHR: aryl hydrocarbon receptor
bHLH-PAS: basic helix-loop-helix Per-Arnt-Sim
KLFs: kruppel-like factors
ncRNAs: non-coding RNAs
CpG: cytosine-phosphate-guanine
AAV: adeno-associated viruses
